# Receptor occupancy of dual glucagon-like peptide 1/glucagon receptor agonist SAR425899 in individuals with type 2 diabetes

**DOI:** 10.1038/s41598-020-73815-5

**Published:** 2020-10-07

**Authors:** Olof Eriksson, Torsten Haack, Youssef Hijazi, Lenore Teichert, Veronique Tavernier, Iina Laitinen, Jan Erik Berglund, Gunnar Antoni, Irina Velikyan, Lars Johansson, Stefan Pierrou, Michael Wagner, Joachim Tillner

**Affiliations:** 1 Antaros Medical AB, Uppsala Science Park, Dag Hammarskjölds Väg 14B, 751 83 Uppsala, Sweden; 2 Science for Life Laboratory, Department of Medicinal Chemistry, Uppsala University, Uppsala, Sweden; 3 R&D Research Platform, Integrated Drug Discovery, Sanofi, Frankfurt, Germany; 4 Translational Medicine, Sanofi, Frankfurt, Germany; 5 R&D Clinical Sciences, Sanofi, Frankfurt, Germany; 6 R&D Clinical Sciences, Sanofi, Montpellier, France; 7 Global Imaging, Sanofi, Frankfurt, Germany; 8 Clinical Trial Consultants AB, Uppsala, Sweden; 9 Department of Medicinal Chemistry, Uppsala University, Uppsala, Sweden; 10 Akademiska Sjukhuset, Uppsala, Sweden; 11 Sanofi-Aventis Deutschland GmbH, Industriepark Höchst, Building H831, 65926 Frankfurt am Main, Germany

**Keywords:** Biomarkers, Endocrinology, Imaging

## Abstract

Unimolecular dual agonists for the glucagon-like peptide 1 receptor (GLP1R) and glucagon receptor (GCGR) are emerging as a potential new class of important therapeutics in type 2 diabetes (T2D). Reliable and quantitative assessments of in vivo occupancy on each receptor would improve the understanding of the efficacy of this class of drugs. In this study we investigated the target occupancy of the dual agonist SAR425899 at the GLP1R in pancreas and GCGR in liver by Positron Emission Tomography/Computed Tomography (PET/CT). Patients with T2D were examined by [^68^Ga]Ga-DO3A-Tuna-2 and [^68^Ga]Ga-DO3A-Exendin4 by PET, to assess the GCGR in liver and GLP1R in pancreas, respectively. Follow up PET examinations were performed after 17 (GCGR) and 20 (GLP-1R) days of treatment with SAR425899, to assess the occupancy at each receptor. Six out of 13 included patients prematurely discontinued the study due to adverse events. SAR425899 at a dose of 0.2 mg daily demonstrated an average GCGR occupancy of 11.2 ± 14.4% (SD) in N = 5 patients and a GLP1R occupancy of 49.9 ± 13.3%. Fasting Plasma Glucose levels (− 3.30 ± 1.14 mmol/L) and body weight (− 3.87 ± 0.87%) were lowered under treatment with SAR425899. In conclusion, SAR425899 demonstrated strong interactions at the GLP1R, but no clear occupancy at the GCGR. The study demonstrates that quantitative target engagement of dual agonists can be assessed by PET.

## Introduction

Unimolecular dual agonists for the glucagon-like peptide 1 receptor (GLP1R) and glucagon receptor (GCGR) are emerging as a potential new class of therapeutics in type 2 diabetes (T2D)^1–3^ and Non-Alcoholic Steato Hepatitis (NASH)^4–6^. This class of drugs aims to combine the effects of agonism at the GLP1R (glucose lowering, decreased appetite) with those of GCGR activation (increased energy expenditure, reduced food intake), thus potentially providing improved glycaemic control in combination with substantial weight reduction. GLP1/glucagon dual agonists have furthermore been shown to ameliorate hepatic fat content^4,5^ and fibrosis^6^ as well as promoting liver regeneration^4^.

Target engagement by the drug is the first step in the effect cascade, thus understanding the relative occupancy to GLP1R versus GCGR is of great importance. The relative effects of induced activation on each of the receptors are likely crucial for optimal efficacy, since for example overly strong activation of GLP1R tends to introduce adverse events (AEs) such as nausea. Overactivation of the GCGR, on the other hand, may increase blood glucose by gluconeogenesis and glycogenolysis, thereby potentially counteracting the improved glycaemic control offered by GLP1R agonism.

Thus, precise and quantitative biomarkers for preclinical and clinical in vivo assessment of engagement at the individual receptors would assist the drug candidate selection and development process. However, such biomarkers are currently lacking, since the pharmacology of the respective receptors are overlapping (i.e. weight loss) or entangled (blood glycemia). Pharmacologically meaningful GLP1R target engagement can still be inferred by some known class effects like reduced post-prandial blood glucose (due to deceleration of gastric emptying) or side effect patterns (e.g. occurrence of nausea or vomiting) at high doses, but there are no similar clear biomarkers for activation of the GCGR in the context of GLP1/glucagon dual agonists.

To provide a tool for direct assessment of occupancy at the GCGR we recently developed the Positron Emission Tomography (PET) radioligand [^68^Ga]Ga-DO3A-Tuna-2 (also known as [^68^Ga]Ga-DO3A-S01-GCG)^7^ that displayed suitable affinity and selectivity for the GCGR in rat, non-human and human liver, in combination and with negligible cross-activity on the GLP1R. The potency of DO3A-Tuna-2 at human recombinant GCGR (EC_50_ 0.4 pM) is comparable to that of native glucagon (EC_50_ 0.5 pM). [^68^Ga]Ga-DO3A-Tuna-2 has been extensively validated as a marker for GCGR occupancy in liver of non-human primate by PET^8^. Previously, we have already developed and validated a similar PET marker for the GLP1R receptor, [^68^Ga]Ga-DO3A-Exendin-4, in non-human primates^9,10^.

SAR425899 was developed for the treatment of patients with T2D and obesity^11–14^ aiming to lower glucose at least similar to marketed GLP-1 agonists, but with superior weight loss over GLP-1 receptor mono-agonists. Preclinical studies with SAR425899 clearly demonstrated dual receptor agonism in vitro and in preclinical in vivo studies, with a higher potency for the GLP1R than for the glucagon receptor in order to counterbalance the GCGR mediated increase of blood glucose levels through GLP-1 agonism^11–13^. Because read outs for GCGR specific binding were missing, data from early clinical studies were unable to confirm the dual agonism of SAR425899 in humans^14^.

The current study was designed to assess the liver GCGR and pancreas GLP1R occupancy of SAR425899 at two different doses (0.12 mg or 0.2 mg daily). Each patient underwent PET examinations with each radioligand both before and after treatment with SAR425899. The occupancy at each receptor was the calculated from the decrease in radioligand binding after treatment compared to baseline^15^.

Here we report the results of a clinical study investigating the GCGR and GLP1R occupancy of SAR425899 in individuals with T2D as assessed by repeated PET imaging.

## Materials and methods

### Clinical trial design

This was a phase Ib, single centre, open-label study (NCT03350191, ClinicalTrials.gov) with 20 days once daily repeated subcutaneous doses of SAR425899 at 2 different dose regimens (A and B) in overweight to obese T2D patients. For Group A, a 3-step dose escalation regimen (Day 1–4: 0.06, Days 5–8: 0.12, Days 9–12: 0.16 and Days 13–20: 0.2 mg, respectively) was chosen, Group B received 0.6 mg for 4 days, and 0.12 mg SAR425899 until Day 20. The 0.2 mg SAR425899 dose was the highest safe dose tested so far in humans and considered the target dose for late stage clinical development. A second lower dose was tested as well to investigate a potential dose dependent effect on receptor binding.

**Figure 1:**
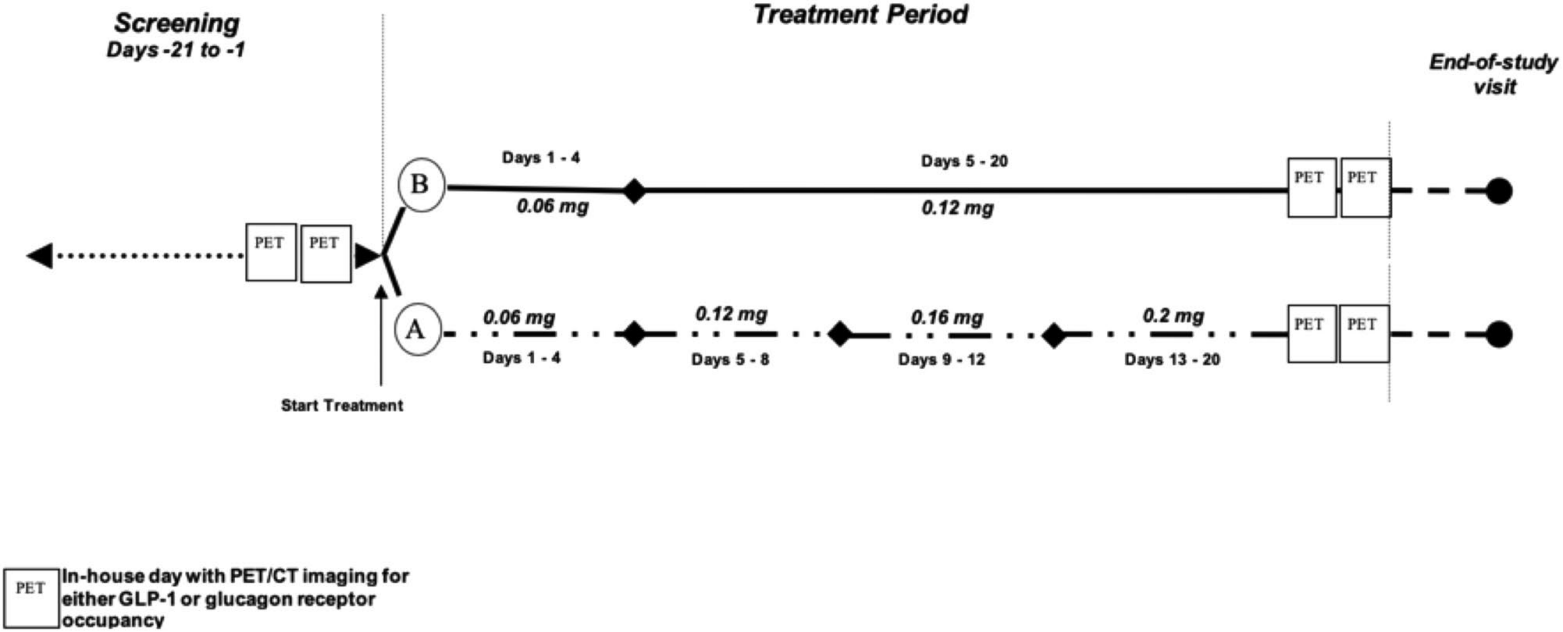
Outline of the study design. All enrolled individuals were assessed by GCGR and GLP1R PET both at baseline and after 3 weeks treatment with SAR425899.

Patients were not randomly assigned to one of the two dose groups. Rather, the investigator aimed for a balanced distribution for gender and BMI class (28–33 and 34–38 kg/m^2^) between both groups. Receptor occupancy of the GCGR and GLP1R was assessed by PET at baseline and at follow-up (Fig. 1). The study was conducted at the site of CTC Clinical Trial Consultants AB, Sweden. All study participants provided written informed consent. Study protocols were approved by the Swedish Medical Products Agency and the Regional Ethical Review Board of Uppsala. The trial was performed in accordance with the guidelines established by the Declaration of Helsinki and the International Conference on Harmonization—Good Clinical Practice.

### Patient population

Eligible participants were men and women aged 18–75 years with a diagnosis of T2D for at least 1 year. Participants were required to have a BMI between 28 and 38 kg/m^2^, to have an HbA1c concentration between 6.5 and 9%. Mild comorbidities were allowed while severe diabetic complications or other diseases were excluded. Otherwise patients were healthy with normal vital signs as assessed by the investigator. Patients were not allowed to be on any antidiabetic medication during the study except for stable metformin and/or sulfonylurea treatment. Pregnant or breast-feeding patients were excluded, in addition pregnancy tests were performed at the time of PET scans to avoid radiotracer injections to undiscovered pregnancies.

The sample size for this trial was based on empirical considerations.

### Pharmacokinetic and pharmacodynamic variables

Blood samples were collected at multiple time points after the last dose on Day 20 for measurement of SAR425899 in plasma using a liquid chromatography with tandem mass spectrometry (LC–MS/MS) method. Peak plasma concentrations (C_max_), time to C_max_ (t_max_) and area under the plasma concentration versus time curve for the last measurable plasma concentration (AUC_0–last_) were calculated using noncompartmental methods.

Body weight and fasting plasma glucose were measured at before breakfast and dosing at baseline and end-of-treatment.

Blood samples for measuring glucagon in plasma were collected shortly before each PET scan with the glucagon tracer only. 2 mL blood was collected in P800 tubes™ (Beckton Dickinson) to prevent proteolytic degradation and glucagon was analyzed using the Mercodia™ ELISA assay.

### Radiochemistry

Good Manufacturing Practise (GMP) grade DO3A-Tuna-2 and DO3A-Exendin-4 were provided by Sanofi. The GMP compliant production of [^68^Ga]Ga-DO3A-Tuna-2 and [^68^Ga]Ga-DO3A-Exendin-4 was developed and conducted on an automated synthesizer (Modular Lab Pharm Tracer, Eckert & Ziegler, Germany) using disposable cassette system. The synthesis of [^68^Ga]Ga-DO3A-Tuna-2 and [^68^Ga]Ga-DO3A-Exendin-4 was developed based on, respectively a manual labelling procedure^7^ and automated procedure^16^ developed earlier. The product formulated in saline containing ethanol (< 10%) was supplied in a sterile glass vial. The radiochemical yield was over 90% with no unknown single impurity of over 5%.

### PET/CT examinations

PET/Computed Tomography (CT) assessment of GCGR and GLP1R availability was performed both at baseline, before start of treatment, and after around 3 weeks of treatment on Day 17 (GLP1R) and Day 20 (GCGR). All assessments were performed 3 h after a standardized meal, to minimize the variability of endogenous hormones (especially GLP1 and glucagon) at the time of each PET assessment.

The baseline assessment of GCGR was performed on a Friday and of the GLP1R on the following Monday. Following treatment, the follow-up PET/CT assessment was performed 3 weeks later in a similar fashion. Prior to each PET/CT examination, individuals were checked for protocol restrictions and exclusion criteria (e.g. female individuals were administered a urine dip stick test to exclude pregnancy).

#### [^68^Ga]Ga-DO3A-Tuna-2

Individuals were positioned in supine position at the bed of a PET/CT scanner (Discovery MI, GE Healthcare, Milwaukee, MI, USA). The individual was positioned with the liver in the 20 cm axial field of view by assistance of a CT scout scan (lateral, 120 kV, 10 mAs).

A low dose CT examination (120 kV, Auto mA 10–30 mA, noise-index 170, rotation-time 0.5 s, full spiral, slice thickness 3.75 mm, pitch 1.53:1) was performed to provide attenuation correction of the PET images as well as anatomical co-registration. Then, 0.5 MBq/kg [^68^Ga]Ga-DO3A-Tuna-2 (0.48 ± 0.03 MBq/kg), corresponding to 0.12 ± 0.06 µg/kg peptide, was administered intravenously as a bolus.

Previous [^68^Ga]Ga-DO3A-Tuna-2 dose finding experiments in non-human primates by PET/CT demonstrated that injected doses of in excess of 0.20 µg/kg DO3A-Tuna-2 tended to begin to saturate the GCGR in the liver^8^. This is a potential issue for all PET tracers targeting receptors or other saturable systems, but especially for highly potent binders.

Thus, it was imperative to keep the administered molar dose of tracer peptide constant and below 0.2 µg/kg in all patients, to avoid any unwanted GCGR saturation of the tracer itself.

Immediately at administration of [^68^Ga]Ga-DO3A-Tuna-2, a 60-min dynamic PET acquisition in list-mode was started. The PET list mode data was reconstructed into 30 frames (12 × 10 s, 6 × 30 s, 5 × 120 s, 5 × 300 s, 2 × 600 s) using an iterative VPFX-S algorithm (three iterations, three subsets, matrix 256 × 256, Z-axis post-filter 3 mm) with all relevant corrections performed.

#### [^68^Ga]Ga-DO3A-Exendin-4

The assessment of GLP1R availability was performed as described above for GCGR. The participant was positioned with the pancreas in the middle of field of view. Approximately 0.5 MBq/kg [^68^Ga]Ga-DO3A-Exendin-4 (0.47 ± 0.03 MBq/kg), corresponding to 0.13 ± 0.04 µg/kg peptide, was administered intravenously as a bolus. The low administered peptide was based on previous dose escalation studies in non-human primates, which demonstrate that DO3A-Exendin-4 doses < 0.15 µg/kg has low to negligible peptide mass effects compared to higher doses^9^.

#### PET data analysis

PET image analysis was performed by the Carimas 2.9 software (Turku PET Center, Turku, Finland). Regions of Interest were segmented on transaxial PET projections assisted by co-registered CT images. In the case of [^68^Ga]Ga-DO3A-Tuna-2, the liver volume was segmented. In the case of [^68^Ga]Ga-DO3A-Exendin-4, the pancreas was segmented, where care was taken to excluded spill-in from the renal cortex uptake. Only voxels fully within each tissue were included to avoid partial volume effects (PVEs). The dynamic PET measurements were expressed as Standardized Uptake Values (SUV) according to Eq. (1)

1 $$ SUV \left( \frac{1}{1} \right) = \frac{{{\raise0.7ex\hbox{${Radioactivity_{tissue } \left( {Bq} \right)}$} \!\mathord{\left/ {\vphantom {{Radioactivity_{tissue } \left( {Bq} \right)} {Volume_{tissue} \left( {cc} \right)}}}\right.\kern-\nulldelimiterspace} \!\lower0.7ex\hbox{${Volume_{tissue} \left( {cc} \right)}$}}}}{{{\raise0.7ex\hbox{${Radioactivity_{injected } \left( {Bq} \right)}$} \!\mathord{\left/ {\vphantom {{Radioactivity_{injected } \left( {Bq} \right)} {Weight_{body} \left( g \right)}}}\right.\kern-\nulldelimiterspace} \!\lower0.7ex\hbox{${Weight_{body} \left( g \right)}$}}}} $$

SUV values for all individual time-frames were evaluated as potential endpoint for binding to the GCGR. Based on this analysis. The SUV value for the timeframe 50–60 min after administration (SUV_55min_) provided the optimal contrast between liver tissue uptake (retention with time) and blood (clearance over time). SUV_55min_ was thus used at endpoint for occupancy calculation. A detailed description of [^68^Ga]Ga-DO3A-Tuna-2 biodistribution including kinetic analysis and dosimetry is outside the scope of the current manuscript and will be reported separately.

During analysis it was observed that treatment with SAR425899 significantly decreased liver volume as well as the plasma glucagon levels. Both factors could potentially quantitatively impact the binding of [^68^Ga]Ga-DO3A-Tuna-2 in the liver. Thus, the SUV_55min_ at follow-up investigation was proportionally corrected for the decrease in liver volume compared to baseline. Additionally, SUV_55min_ at baseline was corrected to the plasma glucagon value of the individual at follow-up to enable direct comparison between the PET assessments.

The correction was performed according to Eq. (2), with pGCG = plasma glucagon level, k = slope of the regression line at baseline of SUV_55min_ (SUV_55min, Baseline_) over plasma glucagon (pGCG_Baseline_)

2 $$ SUV_{55\min , Baseline,pGCG corr} = SUV_{55\min , Baseline} + k \times (pGCG_{{Follow{\text {-}}up}} - pGCG_{Baseline} ) $$

Estimation of slope k was based on all n = 13 subjects for whom SUV_55min_ and plasma glucagon values were available at baseline.

The SUV_55min_ assessment of each tracer is a surrogate marker of the amount of available receptors in each tissue (GCGR in liver, GLP1R in pancreas). Thus, the decrease in PET tracer binding in each tissue is proportional to the occupancy of the study drug. The occupancy at each receptor is calculated according to Eq. (3)

3 $$ Occupancy \left( \% \right) = 100 - \left( {\frac{{SUV_{55\min , Follow{\text {-}}up} }}{{SUV_{55\min , Baseline} }}} \right) \times 100 $$

#### Statistical considerations

Data were analyzed exploratory and reported as averages and standard deviations (SD) in the form of Arithmetic mean (SD). As only one patient completed the study in Group B, the two groups were pooled for safety analyses. Pharmacodynamics and pharmacokinetics were analyzed per last SAR425899 dose received (0.12 or 0.2 mg).

## Results

### Patient population

Thirteen T2D patients were enrolled and received at least one dose of SAR425899. Seven patients were treated according to protocol and completed the entire study period including follow-up examination with both PET tracers. Six patients prematurely discontinued the study. Six out of 7 completers were included in the final evaluation for receptor occupancy, one patient had a major protocol deviation (dosing titration irregularity) and was excluded from this analysis. The number of evaluable completers was 5 for 0.2 mg SAR425899 and 1 for 0.12 mg SAR425899.

Mean (SD) age, weight and BMI at baseline of N = 13 enrolled patients was 65.9 (8.8) years, 97.8 (8.09) kg and 31.4 (3.0) kg/m^2^, respectively, for the. One female subject was included only.

### Safety and tolerability

The most frequently observed adverse events (AEs) following start of treatment were gastrointestinal disorders observed in 10 of 13 patients (Table 1). AEs reported most frequently were decreased appetite and nausea in seven patients each, fatigue in four patients, headache and eructation in three patients each, and constipation, dyspepsia, gastritis, vomiting, and malaise in two patients. One patient experienced a Grade 3 pyelonephritis, at a dose level of 0.06 mg. Treatment was stopped, and the patient was permanently withdrawn from the study. The Investigator considered the event not related to treatment with SAR425899 or the study procedures. Overall, 6 out of 13 patients prematurely discontinued the study, all of them because of an AE (lipase increased, pyelonephritis, Grade 2 discomfort, constipation, nausea, presyncope).

**Table 1:** Adverse events (AE).

Primary system organ class Preferred term [n (%)]	SAR425899 (N = 13)
Any class	12 (92.3%)
**Infections and infestations**	1 (7.7%)
Pyelonephritis	1 (7.7%)
**Metabolism and nutrition disorders**	7 (53.8%)
Decreased appetite	7 (53.8%)
**Psychiatric disorders**	1 (7.7%)
Anxiety	1 (7.7%)
**Nervous system disorders**	4 (30.8%)
Headache	3 (23.1%)
Dizziness postural	1 (7.7%)
Presyncope	1 (7.7%)
**Respiratory, thoracic and mediastinal disorders**	2 (15.4%)
Hiccups	1 (7.7%)
Oropharyngeal pain	1 (7.7%)
**Gastrointestinal disorders**	10 (76.9%)
Nausea	7 (53.8%)
Eructation	3 (23.1%)
Constipation	2 (15.4%)
Dyspepsia	2 (15.4%)
Gastritis	2 (15.4%)
Vomiting	2 (15.4%)
Abdominal pain	1 (7.7%)
Diarrhoea	1 (7.7%)
Dry mouth	1 (7.7%)
Gastrointestinal disorder	1 (7.7%)
**Musculoskeletal and connective tissue disorders**	1 (7.7%)
Arthralgia	1 (7.7%)
**General disorders and administration site conditions**	8 (61.5%)
Fatigue	4 (30.8%)
Malaise	2 (15.4%)
Asthenia	1 (7.7%)
Discomfort	1 (7.7%)
Early satiety	1 (7.7%)
**Investigations**	1 (7.7%)
Lipase increased	1 (7.7%)

*N* number of patients treated within the group, *n (%)* number and % of patients with at least one AE in each category.

### Pharmacokinetics and exposure

All seven completers having undergone PET assessments at the end of treatment were exposed to SAR425899 plasma levels comparable to other clinical studies having applied similar dose levels. On Day 20 following administration of the 0.2 mg SAR425899 dose regimen, geometric mean values for SAR425899 AUC_0–last_ and C_max_ were 217 ng h/mL and 25.1 ng/mL, respectively, and the median t_max_ was 7.62 h.

### Pharmacodynamics

**Figure 2:**
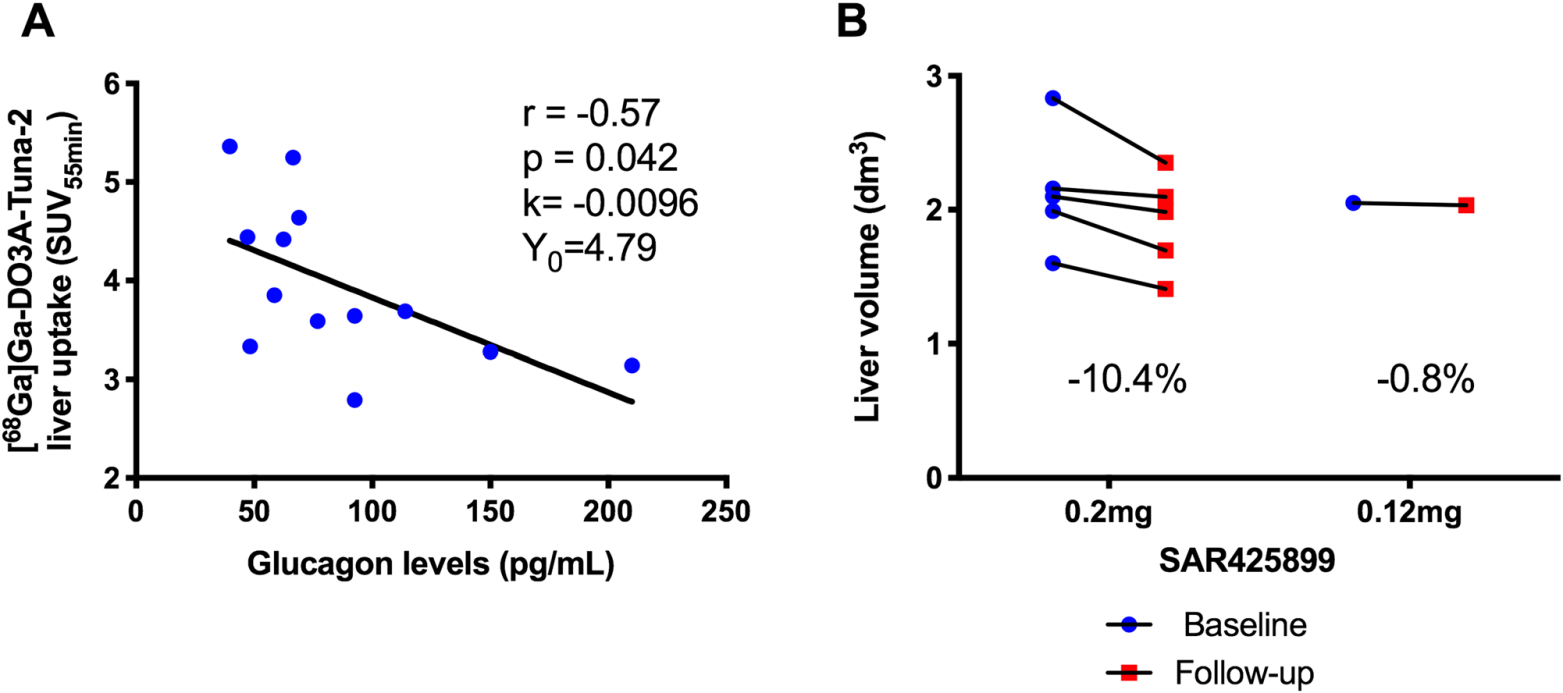
Correlation between [^68^Ga]Ga-DO3A-Tuna-2 liver uptake (SUV_55min_) and plasma glucagon levels in individual patients at the baseline PET/CT examination (n = 13) (**A**). Decrease in liver volume during treatment with SAR425899 (**B**).

Fasting Plasma Glucose (FPG) levels were lowered under treatment with SAR425899. Mean change from baseline (SD) for 0.2 mg SAR425899 on Day 20 was − 3.30 (1.14) mmol/L. Mean change from baseline for body weight on Day 20 at a dose of 0.2 mg SAR425899 was − 3.87% (0.87). Glucagon concentrations in plasma decreased under treatment with SAR425899 although the variability was high. Mean change from baseline (SD) was − 52.23 ng/L (45.58) between baseline and Day 17 at a dose of 0.2 mg SAR425899. Furthermore, there was a negative correlation between the liver uptake of [^68^Ga]Ga-DO3A-Tuna-2 and the glucagon levels, indicating competition for the GCGR (Fig. 2A). Liver volume values decreased under treatment with SAR425899 by on average 10.4 (6.1) % with baseline values of 2.14 (0.45) dm^3^ and follow up values of 1.91 (0.36) dm^3^ (Fig. 2B).

### GCGR occupancy

**Figure 3:**
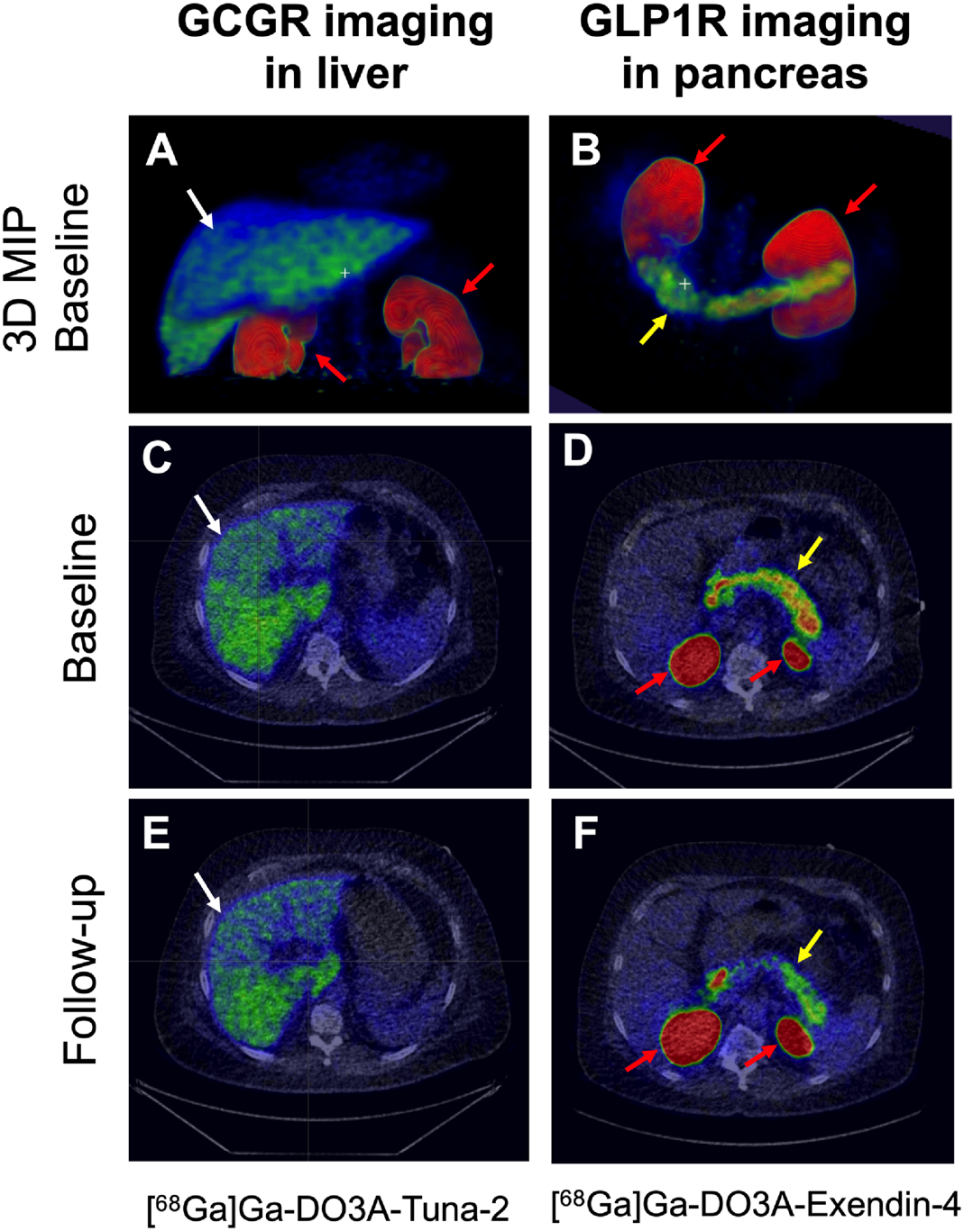
Representative images (**A**, **B**: 3D Maximal Intensity Projections (MIP) and **C**–**F**: trans-axial projection) of PET uptake for the tracer for the GCGR (left panels, **A**, **C**, **E**) and GLP1R (right panels, **B**, **D**, **F**). [^68^Ga]Ga-DO3A-Tuna-2 displayed strong binding in the liver which is rich in GCGR combined with low or negligible uptake in surrounding GCGR negative tissues (**A**, **C**). [^68^Ga]Ga-DO3A-Exendin-4 on the other hand showed strong binding to GLP1R in pancreas at baseline (**B**, **D**). Both PET tracers were excreted through renal route and trapped in the kidney cortex. The reduction in binding at the follow up examinations following treatment are proportional to the SAR425899 drug occupancy at the respective receptor (**E**, **F**). The baseline and follow-up images for the respective tracers are normalized to SUV = 5 and are directly comparable. White arrows indicate liver, yellow arrows indicate pancreas and red arrows indicate kidneys.

**Figure 4:**
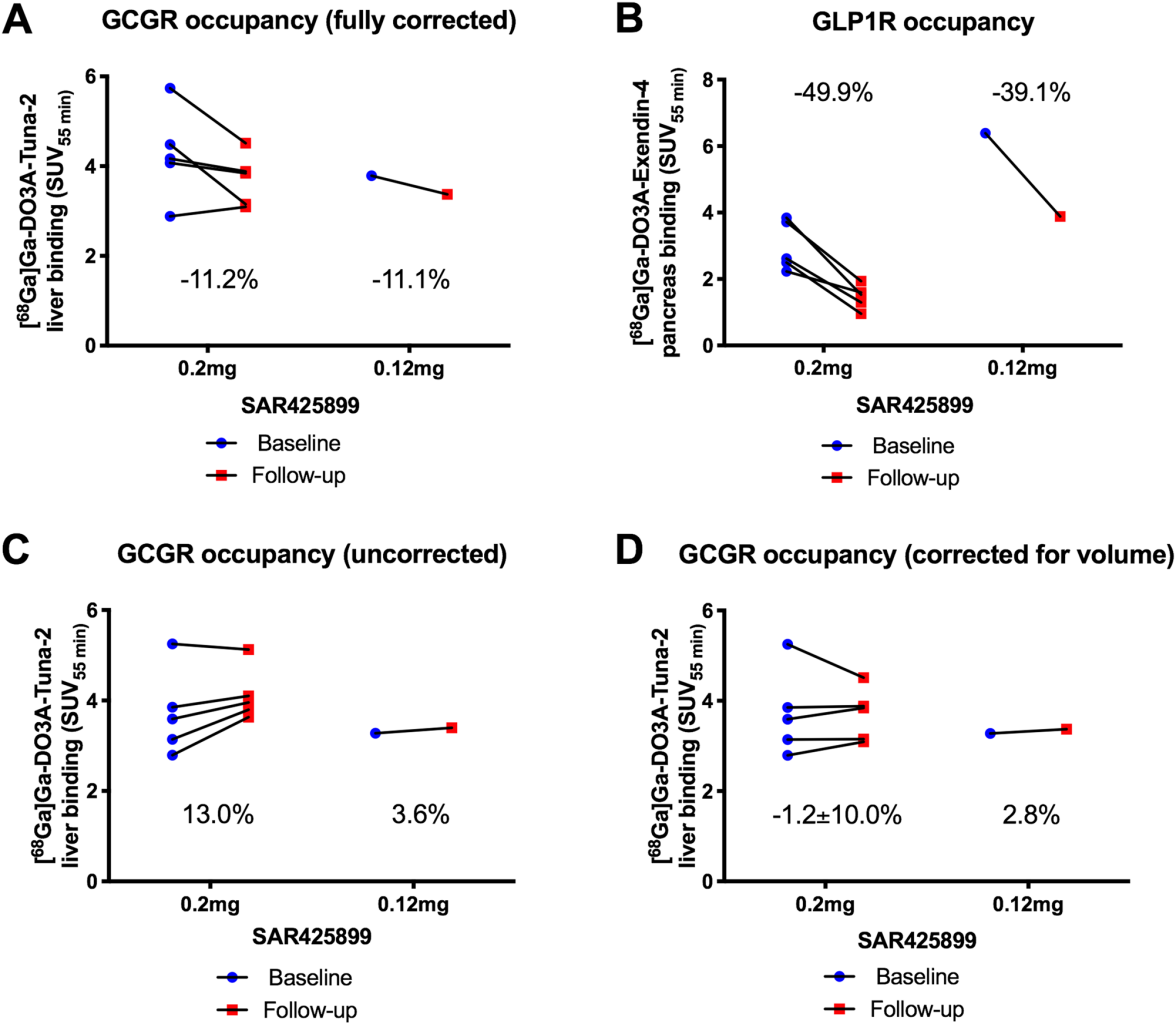
Results of the PET assessments. Blue circles represent baseline PET examinations and red squares represent follow-up PET examinations on treatment with 0.2 or 0.12 mg SAR425899. Liver binding of [^68^Ga]Ga-DO3A-Tuna-2, corrected for plasma glucagon levels and decrease in liver volume, at baseline and on SAR425899 treatment (**A**). [^68^Ga]Ga-DO3A-Exendin-4 binding in pancreas at baseline and follow-up (**B**). The uncorrected [^68^Ga]Ga-DO3A-Tuna-2 liver binding (**C**) and following correction only for the change in liver volume (**D**) is also shown for clarity of the analysis. Percentages indicate change in binding, i.e. occupancy of SAR425899 treatment compared to baseline.

GCGR PET tracer [^68^Ga]Ga-DO3A-Tuna-2 demonstrated strong liver binding in all baseline examinations (Fig. 3). The liver binding, corrected for decreases in liver volume and plasma glucagon levels, decreased from SUV_55min_ 4.27 (1.02) at baseline to SUV_55min_ 3.70 (0.59) at follow up (Fig. 4A). This corresponded to an average (SD) GCGR occupancy of 11.2 (14.4) % at a dose of 0.2 mg SAR425899. The individual receptor occupancy values at the GCGR ranged between − 7.2 to 29.6% (n = 5). The GCGR occupancy at a dose of 0.12 mg SAR425899 was 11.1% (n = 1). The uncorrected [^68^Ga]Ga-DO3A-Tuna-2 liver binding (Fig. 4C) and following correction only for the change in liver volume (Fig. 4D) is also shown for transparency.

### GLP1R occupancy

All baseline examinations with [^68^Ga]Ga-DO3A-Exendin-4 demonstrated strong uptake in the pancreas (Fig. 3). Mean (SD) [^68^Ga]Ga-DO3A-Exendin-4 binding in pancreas uptake was decreased from SUV_55min_ 2.98 (0.74) at baseline to SUV_55min_ 1.46 (0.37) at a dose of 0.2 mg SAR425899 (Fig. 4B). The average (SD) occupancy rate on the GLP-1 receptor by 0.2 mg SAR425899, was estimated as 49.9 (13.3) % (n = 5). At the dose of 0.12 mg SAR425899, the occupancy was 39.1% (n = 1). The inter-individual variability of receptor occupancy was acceptable.

## Discussion

The target engagement and receptor occupancy of SAR425899, a compound for which GLP1R and GCGR binding has been shown in preclinical settings, was for the first time quantitatively assessed in human. This was also the first study having investigated a PET tracer targeting the GCGR in humans.

The planned number of 12 subjects (six per each dose group A and B) having completed SAR425899 treated and evaluated for PET receptor occupancy was not achieved in this study. The main reason for this was a high drop out rate of 6 out of 13 subjects enrolled because of AEs. It was therefore decided during the study to fill up the 0.2 mg dose group (A) first to get six evaluable subjects at this dose. When this goal was achieved a preliminary analysis of PET data for this dose group showed a very low binding of SAR425899 to the GCCR, suggesting that a dose of 0.12 mg will not provide further significant occupancy data. It was then decided to stop further recruitment and the study was discontinued.

The AE pattern observed in this study was comparable with other GLP-1 agonists, with AEs related to gastrointestinal disorders reported more frequently. The incidence of withdrawals because of an AE, however, was higher than other drugs of this class and also compared to results from other studies with SAR425899^14^. Three participants prematurely withdrawn reported an AE related to a gastrointestinal disorder (lipase increase, nausea, constipation), a known class effect for GLP-1 agonists. Usually this is circumvented by carefully increasing the dose, suggesting the dose escalation regimen for SAR425899 was inappropriately selected for this study. The occupancy at the GCGR and GLP1R was thus assessed in the patients without significant AEs. The evaluable completers in this study therefore likely represent the intended treatment population, e.g. overweight to obese patients with T2D tolerating the dose regiment.

In addition to the AE pattern SAR425899 also displayed several pharmacological effects known to be exerted by GLP-1 agonists, including improved glycemic control and weight loss. These data were in line with previous clinical studies^14^, confirming sufficient SAR425899 drug exposure at dose of 0.2 mg and GLP-1 receptor activation.

Marked weight loss and glucose lowering, as observed in this study, may have rapid and meaningful effects on the hepatic volume in short time periods, due primarily to the depletion of liver glycogen, which in turn bind large amounts of water. A reduction of the liver volume due to decrease in glycogen and water could likely affect the PET data by increasing the hepatocyte density (and in turn the GCGR density) per voxel. The liver volume was therefore assessed on CT images, and the [^68^Ga]Ga-DO3A-Tuna-2 liver uptake SUV_55min_ data corrected for the corresponding change in hepatic volume on an individual level. However, this correction may also introduce errors especially since there are inhomogeneities in GCGR expression levels in different parts of the liver (Fig. 3A,C,E). Furthermore, this correction assumes negligible changes on GCGR expression on hepatocytes over time. The occupancy data before the different corrections are therefore reported separately in Fig. 4C,D. Further corrections regarding the potentially non-isotropic GCGR expression could be attempted, but regardless the impact on the occupancy is expected to be low, while potentially introducing noise to the assessment. [^68^Ga]Ga-DO3A-Exendin-4 uptake in pancreas is not expected to require correction, as no similar mechanism for rapid volume change is present in pancreas.

[^68^Ga]Ga-DO3A-Tuna-2 binding to GCGR is assumed to be sensitive to endogenous levels of glucagon at the time of the examination, since both share the same binding^7^. Accordingly, a significant increase in glucagon levels could compete with [^68^Ga]Ga-DO3A-Tuna-2 for the GCGR, presented as an apparent decrease in [^68^Ga]Ga-DO3A-Tuna-2 binding. Conversely, a *decrease* in glucagon levels between baseline and follow-up assessment could present itself as an apparent *increase* in [^68^Ga]Ga-DO3A-Tuna-2 binding due to reduced competition for binding. Although conditions around the time of PET examinations were kept as stable as possible, glucagon levels were lower at follow-up compared to baseline. As outlined above, this could mask a true decrease in GCGR availability. Accordingly, this effect was observed as negative linear correlation between [^68^Ga]Ga-DO3A-Tuna-2 liver binding and the glucagon concentration in the baseline data set (Fig. 2A). Thus, the SUV_55min_ at follow-up was corrected to the glucagon value at baseline to enable direct comparison.

SUV_55min_ (e.g. SUV at the 55 min time point) was here chosen as a surrogate marker for receptor density of GCGR and GLP1R. PET has historically been widely used for drug occupancy studies in the brain specifically^15^. The brain physiology entails simplification of some aspects of the occupancy calculation, which are not validated in peripheral tissue such as here. These include for example arterial plasma concentration as an input signal for compartmental or graphical modeling of radioligand/target receptor interactions such as Binding Potential (BP), a well understood and validated vascular contribution to the PET signal in tissue as well as the possibility of using brain regions without receptor expression as reference tissue. For peripheral tissues such as liver and pancreas, it is difficult to identify reference tissues (which should exhibit similar perfusion and tracer delivery, but without receptor expression). Additionally, the liver has a dual supply of blood from both arterial and venous sources (approximately by a 30/70 ratio) which makes estimation of an input signal for PET kinetic modelling challenging. The relative contribution of arterial and venous blood to the liver is also affected by vascular disease, for which patients with T2D are at risk. Finally, therapeutic intervention which ameliorate T2D (e.g. such as dual agonists) could theoretically normalize the arterial/venous ratio for liver blood supply. With these issues in mind, and the potential errors the kinetic modeling of the peripheral tissues may introduce, we decided to use SUV_55min_ for the occupancy calculations.

The mean (SD) receptor occupancy for the GLP-1 receptor in the pancreas obtained for SAR425899 at a dose of 0.2 mg was 49.9 (13.3)%. These data are line with pharmacodynamic results from other studies with SAR425899^14^, confirming the drug to be a potent GLP-1 receptor agonist.

**Figure 5:**
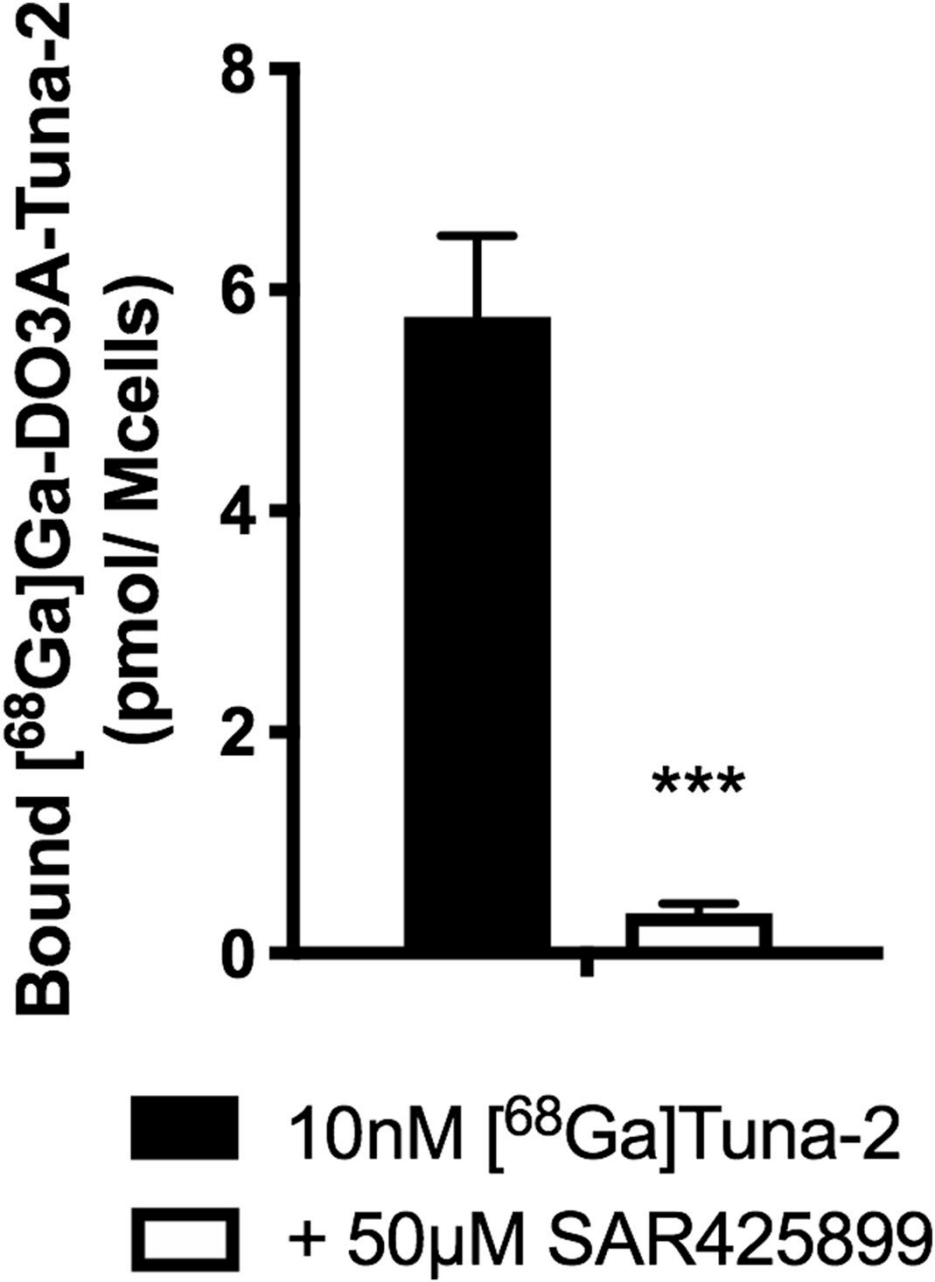
[^68^Ga]Ga-DO3A-Tuna-2 was displaced by SAR525899 in vitro. HEK293 cells overexpressing the human GCGR were incubated with either [^68^Ga]Ga-DO3A-Tuna-2 alone, or following pre-treatment with 50 µM SAR525899. Data from three independent repeated experiments. Stars indicate p < 0.001 as assessed by a Students t-test.

However, the mean receptor occupancy value for the GCGR at the same dose was 11.2 (14.4)% only, indicating no clear occupancy of the GCGR in the liver by SAR425899 in this study. The reason for this low receptor binding capacity is unknown. In vitro receptor binding studies data have shown SAR425899 to bind to the GCGR^11,14^. Similarly, SAR425899 competes for the same binding site as [^68^Ga]Ga-DO3A-Tuna-2 in vitro (Fig. 5), suggesting that SAR425899 interactions with GCGR in vivo should be detected by the PET assessment.

Noteworthy, the in vitro binding affinity for SAR425899 at the GLP1R was ~ fivefold higher than at the GCGR. This ratio of binding capacities has been chosen to prevent unwanted glucagon effects like increase of blood glucose. In fact, an increase of blood glucose has not been observed so far in a clinical setting. Rather known effects associated with GLP1R binding were detected at a dose of 0.2 mg SAR425899, including reduction of body weight, HbA1c, FPG and PPG but also the occurrence of gastrointestinal disorders. The exposure achieved with this dose in humans, however, might be too low to reach a sufficiently high GCGR occupancy as observed in this study. Further mechanistic studies were initiated investigating whether SAR425899 targets the GCGR including effects on energy expenditure and amino acid patterns.

Limitations of the study are acknowledged including, but not limited to, small sample size due to high dropout rate, combined with high variability of GCGR occupancy, unknown changes in GCGR density and the relevance of low receptor occupancy in terms of clinical efficacy. Further studies are required to fully elucidate the potential of the glucagon tracer presented here as a valuable tool for receptor target engagement and drug development.

In conclusion, SAR425899 demonstrated strong binding to the GLP1R, but low occupancy at the GCGR. The study demonstrated for the first time that quantitative target engagement can be assessed at the GLP1R and GCGR by PET.
